# Smad Nuclear Interacting Protein 1 Acts as a Protective Regulator of Pressure Overload‐Induced Pathological Cardiac Hypertrophy

**DOI:** 10.1161/JAHA.116.003943

**Published:** 2016-10-26

**Authors:** Yu‐yan Lu, Da‐chun Xu, Yi‐fan Zhao, Guo‐fu Zhu, Meng‐yun Zhu, Wei‐jing Liu, Xue‐jing Yu, Wei Chen, Zheng Liu, Ya‐wei Xu

**Affiliations:** ^1^ Department of CardiologyCardiovascular Disease InstituteShanghai Tenth People's HospitalTongji University School of MedicineShanghaiChina

**Keywords:** cardiac hypertrophy, heart failure, nuclear factor‐κB, signaling pathway, Smad nuclear interacting protein 1, Hypertrophy

## Abstract

**Background:**

Smad nuclear interacting protein 1 (SNIP1) plays a critical role in cell proliferation, transformation of embryonic fibroblasts, and immune regulation. However, the role of SNIP1 in cardiac hypertrophy remains unclear.

**Methods and Results:**

Here we examined the role of SNIP1 in pressure overload–induced cardiac hypertrophy and its mechanisms. Our results demonstrated that SNIP1 expression was downregulated in human dilated cardiomyopathic hearts, aortic banding‐induced mice hearts, and angiotensin II–treated cardiomyocytes. Accordingly, SNIP1 deficiency significantly exacerbated aortic banding–induced cardiac hypertrophy, fibrosis, and contractile dysfunction, whereas cardiac‐specific overexpression of SNIP1 markedly recovered pressure overload–induced cardiac hypertrophy and fibrosis. Besides that, SNIP1 protected neonatal rat cardiomyocytes against angiotensin II–induced hypertrophy in vitro. Moreover, we identified that SNIP1 suppressed nuclear factor‐κB signaling during pathological cardiac hypertrophy, and inhibition of nuclear factor‐κB signaling by a cardiac‐specific conditional inhibitor of κB^S^ ^32A/S36A^ transgene blocked these adverse effects of SNIP1 deficiency on hearts.

**Conclusions:**

Together, our findings demonstrated that SNIP1 had protective effects in pressure overload–induced pathological cardiac hypertrophy via inhibition of nuclear factor‐κB signaling. Thus, SNIP1 may be a novel approach for the treatment of heart failure.

## Introduction

Cardiac hypertrophy is an adaptive process of cardiac muscle in response to diverse biomechanical loads, such as hypertension, endocrine imbalance, and myocardium infarction.^1^, ^2^ Enduring hypertrophy is followed by increased cardiac fibrosis, cell death, and contractile dysfunction, eventually developing into heart failure.^3^

Recently, it has been proven that several transcription regulators such as nuclear factor‐κB (NF‐κB), GATA transcription factors, nuclear factor of activated T cells, myocardium‐related factor A, and myocyte enhancer factor‐2 were activated in development of cardiac hypertrophy, which could induce the expression of hypertrophic genes promoting pathological cardiac hypertrophy.^2^, ^4^, ^5^, ^6^, ^7^, ^8^, ^9^, ^10^ Although numerous studies of cardiac hypertrophy have been reported, the molecular mechanisms of cardiac hypertrophy remain to be elucidated. Of note, among these transcription regulators, NF‐κB signaling plays a pivotal role. In canonical NF‐κB signaling, the NF‐κB dimer including p65 and p50 activity is inhibited in the cytoplasm by inhibitor of NF‐κB α (IκBα), which is phosphorylated and degradated after activation of the inhibitor of κB kinase (IKK) complex. During the process, NF‐κB is translocated from cytoplasm to nucleus, leading to target gene activation, such as hypertrophic genes and inflammatory factors, which in turn further activate the canonical NF‐κB signaling pathway.^11^, ^12^

Smad nuclear interacting protein 1 (SNIP1), as a 396‐amino acid nuclear protein, is an evolutionarily conserved protein containing a bipartite nuclear localization signal and a Forkhead‐associated domain. Further studies indicated that SNIP1 could inhibit p300‐dependent transforming growth factor‐β signal transduction.^13^ Biologically, SNIP1 could regulate cell proliferation and transformation of embryonic fibroblasts associated with c‐Myc; furthermore, reduction of SNIP1 resulted in an obvious decrease of cells in the G1 phase of the cell cycle and inhibition of cyclin D promoter activity.^14^, ^15^ Kim et al also reported that SNIP1 could suppress NF‐κB signaling dependent on CBP/p300 transcriptional co‐activators.^16^ However, the role of SNIP1 in cardiomyocytes remodeling and pressure overload–induced cardiac hypertrophy still remains unknown.

Based on the aforementioned studies and cardiomyocytes without the ability of proliferation and transformation, we hypothesized that SNIP1 might inhibit NF‐κB signaling to protect cardiomyocytes from hypertrophic stimulations, especially chronic pressure overload. Our data as shown here clarify a crucial role for cardiac‐specific SNIP1 in the protective effects of cardiomyocytes by NF‐κB signaling suppression.

## Materials and Methods

### Reagents

Antibodies against the following proteins were purchased from Santa Cruz Biotechnology (Dallas, TX): atrial natriuretic peptide (ANP, sc20158 1:200), β‐myosin heavy chain (β‐MHC, sc53090 1:200), and SNIP1 (sc47929 1:200). Antibody against phospho‐IKKβ (ab59195 1:500) was purchased from Abcam Biochemicals (Cambridge, MA). The following antibodies were purchased from Cell Signaling Technology (Danvers, MA): phospho‐NF‐κB p65 (#3033 1:1000), total‐NF‐κB p65 (#4764 1:1000), total‐IKKβ (#8943 1:1000), phospho‐IκBα (#2859 1:1000), total‐IκBα (#4812 1:1000), cleaved caspase‐3 (#9661 1:1000), and GAPDH (#2118 1:1000). Fetal bovine serum was purchased from Gibco (Grand Island, NY). The other reagents for cell culture were purchased from Sigma (St. Louis, MO).

### Human Samples

In accordance with the principle outlined in the Declaration of Helsinki, all of the procedures involving human heart samples were conducted and approved by the Ethics Committee at Tongji University. The human dilated cardiomyopathic hearts were obtained from patients after a heart transplantation operation. The donor human hearts were obtained from patients who died of noncardiac diseases or died from accidents, which were unsuitable for heart transplantation technically. Written informed consent was obtained from each dilated cardiomyopathy patient and the relatives of the heart donors.

### Generation of Animal Models

All of the animal procedures were approved by the Animal Care and Use Committees of Shanghai Tenth People's Hospital.

### Generation of Global SNIP1 Knockout Mice

Global SNIP1 knockout mice were generated by CRISPR‐Cas9 techniques. The single guiding RNA targeting mouse SNIP1 gene was first predicted by an online CRISPR design tool (http://crispr.mit.edu). Then, a pair of oligomers (oligo1: TAACGGTCGTCAGAGGCGTCGCCTGAC; oligo2: AAACGTCAGGCGACGCTCTGACGACC) were annealed and insert into the BsaI restriction site of pUC57‐sgRNA expression vector (Addgene 51132). Primers containing the T7 promoter and the sgRNA region (Forward primer: GATCCCTAATACGACTCACTATAG; Reverse primer: AAAAAAAGCACCGACTCGGT) were designed to amplify the DNA by polymerase chain reaction. MEGAshortscript Kit (Amibion, AM1354) and miRNeasy Micro Kit (Qiaen, 217084) was used to transcribe and purify the sgRNA sequence, respectively. The Cas9 plasmid (Addgene 44758) was transcribed in vitro using the T7 Ultra Kit (Ambion, AM1345). Cas9 mRNA was purified by the RNeasy Mini Kit (QiaGen, 74104) following the manufacturer's instructions. MRNA of Cas9 and sgRNA were both injected into 1‐cell embryos using the FemtoJet 5247 microinjection system. Primers SNIP1‐131‐F (5′‐GGACCGTGCAAGTGACTCTC‐3′) and SNIP1‐131‐R (5′‐TCAGACGCTCCTGTTTCACC‐3′) were used to verify the mutation of F1 and F2 offspring. The wild‐type allele contained a 131‐bp amplicon and the mutant allele contained a 109‐bp amplicon. The protein level of SNIP1 was analyzed by Western blot.

### Generation of Cardiac‐Specific SNIP1 Overexpression Mice

First, the lacZ gene in pCAG‐loxP‐CAT‐loxP‐lacZ was replaced by full‐length mouse SNIP1 cDNA to generate a construct of pCAG‐loxP‐CAT‐loxP‐SNIP1. Then, this construct was linearized and purified with the QIAquick Gel Extraction Kit (Qiagen, 28704) following the manufacturer's instruction. After that, the vector containing the construct was microinjected into fertilized murine embryos to obtain the transgenic mice. Tail genomic DNA from the founder transgenic mice was used to identify the F0 offspring. The primers for identification were CAG‐Forward (5′‐CCCCCTGAACCTGAAACATA‐3′) and SNIP1‐Reverse (5′‐TCCGTGGTGACTTGCTTCTT‐3′). The size of the amplification products was expected as 595 bp. After identification, the transgenic mice were bred with C57BL/6J background mice to get F1 and F2 offspring. To induce cardiac‐specific SNIP1 overexpression, the CAG‐loxP‐CAT‐loxP‐SNIP1 transgenic mice were crossed with transgenic mice that carried Cre genes downstream of the α‐MHC gene promoter (Jackson Laboratory, 005650) to generate CAG‐CAT‐mSNIP1/α‐MHC‐MerCreMer double transgenic mice. Cre‐mediated recombination of floxed alleles was induced by intraperitoneal injection of tamoxifen (80 mg/kg per day, Sigma, T‐5648) for 5 consecutive days at 6 weeks old to obtain cardiac‐specific SNIP1 conditional transgenic (SNIP1‐TG) mice. Four independent transgenic lines were successfully generated. The CAG‐CAT‐mSNIP1/α‐MHC‐MerCreMer mice without tamoxifen injection (CSMC) were used as the control group.

### Generation of IκBα^S32A/S36A^‐TG/SNIP1‐Knockout Mice

C57BL/6J background IκBα^S32A/S36A^‐TG mice were kindly provided by Professor Hongliang Li.^11^ We crossed SNIP1 knockout mice with IκBα^S32A/S36A^‐TG mice to produce the IκBα^S32A/S36A^‐TG/SNIP1 heterozygous mice, which were then crossed back with SNIP1 knockout mice to obtain IκBα^S32A/S36A^‐TG/SNIP1‐knockout mice. Western blot was used to identify the expression of SNIP1 and IκBα.

### Aortic Banding Operation

Male mice 8 to 10 weeks of age were subjected to an aortic banding (AB) operation, as described in detail previously.^1^ In brief, the left chest was opened and the descending thoracic aorta was identified after mice were anesthetized by intraperitoneal injection of sodium pentobarbital (50 mg/kg, Sigma). Then, the descending thoracic aorta was ligated with 7–0 silk sutures by a 27‐ or 26‐gauge needle, and the needle was removed immediately after ligation. Finally, antibiotics were used before the thoracic cavity was closed. The same procedure was performed in the sham‐operated mice, only without aortic ligation. Mice were euthanized and the tissues were weighed 4 and 8 weeks later after operation.

### Echocardiography

MyLab 30CV ultrasound (Biosound Esaote Inc) was used for echocardiography as previously described.^1^, ^11^ After mice were anesthetized with 1.5% to 2% isoflurane, the parameters of mouse heart were measured from parasternal short‐axis view and parasternal long‐axis view at a frame rate of 120 Hz. Left ventricular (LV) end‐systolic diameter (LVDs) and LV end‐diastolic diameter (LVDd) were measured from the LV M‐mode tracing with a sweep speed of 50 mm/s at the midpapillary muscle level. Fractional shortening (FS) was calculated by the formula: FS=(LVDd−LVDs)/LVDd×100%.

### Histological Analysis

Hearts were excised and immediately placed in a 10% potassium chloride solution to guarantee that the hearts were arrested in diastole. Then, the hearts were fixed for more than 24 hours in 10% formalin, dehydrated, and embedded in paraffin. Subsequently, the hearts were transversely sectioned at 5 μm when the section is close to the left and right ventricles. Sections of each sample were stained with hematoxylin–eosin (HE) and picrosirius red following standard procedures. Furthermore, sections were stained with fluorescein isothiocyanate–conjugated wheat germ agglutinin (WGA, Invitrogen Corp) to confirm the results observed in HE staining (WGA for cell membrane and DAPI for nuclei). We measured the cross‐sectional area (HE) and LV collagen volume (picrosirius red) by Image‐Pro Plus 6.0. More than 100 LV cardiomyocytes’ cross‐sectional areas and more than 25 fields were measured in each group.

### Neonatal Rat Cardiomyocytes Culture and Recombinant Adenoviral Vectors

As previously described,^1^ neonatal rat cardiomyocytes (NRCMs) were isolated from 1‐ to 2‐day‐old Sprague–Dawley neonatal rats. First, rats were euthanized by swift decapitation and the hearts were immediately excised and minced. Then hearts were digested in PBS containing 0.03% trypsin and 0.04% collagenase type II. Finally, after removing the fibroblast using a differential attachment technique, NRCMs were seeded at a density of 1×10^6^ cells/well onto 6‐well culture plates coated with gelatin in DMEM/F12 medium supplemented with 20% fetal bovine serum, bromodeoxyuridine (0.1 mmol/L, to inhibit the proliferation of cardiac fibroblasts), and penicillin/streptomycin. Two days later, the culture medium was replaced to serum‐free DMEM/F12 for 12 hours prior to stimulation with angiotensin II (Ang II, 100 nmol/L)^1^, ^17^ or PBS, which was administered for 24 and 48 hours to induce cardiomyocyte hypertrophy.

NRCMs overexpressing SNIP1 were generated by incubation with the replication‐defective adenoviral vectors, by which the entire coding region of rat SNIP1 gene under the control of the cytomegalovirus promoter was encompassed (AdSNIP1). A similar adenoviral vector encoding the GFP gene (AdGFP) was used as a control. In addition, the rat AdshSNIP1 was used to knock down the SNIP1 expression in NRCMs, and AdshRNA was used as the nontargeting control. The NRCMs were infected with AdGFP, AdSNIP1, AdshRNA, and AdshSNIP1 in diluted media at a multiplicity of infection of 100 for 24 hours.

### Immunofluorescence Analysis

The NRCMs were stained with α‐actinin antibody to determine cell surface areas by immunofluorescence staining. Following standard immunofluorescence staining procedures, NRCMs were permeabilized with 0.1% Triton X‐100 in PBS for 40 minutes after infection with different adenoviruses for 24 hours. Then, the NRCMs were stained with α‐actinin (Sigma‐Aldrich, A7811, 1:100 dilution), and visualized using a fluorescence microscope (Olympus, Tokyo, Japan). Finally, the cell surface areas of NRCMs were measured using Image‐Pro Plus 6.0. More than 50 cells were measured in each experimental group.

The heart sections were double‐stained with SNIP1 and α‐actinin antibodies to investigate the expression of SNIP1 in the control and failing hearts. In addition, we assessed the role of SNIP1 in apoptosis by TUNEL staining using an ApopTag^®^ Plus In Situ Apoptosis Fluorescein Detection Kit (S7111; Millipore), according to the manufacturer's protocol.

### Quantitative Real‐Time Polymerase Chain Reaction

After total mRNA from mice ventricular tissues or NRCMs was extracted by TRIzol (15596‐026, Invitrogen), cDNA was instantly cloned using reverse transcription polymerase chain reaction by the Transcriptor First Strand cDNA Synthesis Kit (04896866001; Roche, Basel, Switzerland). Selected gene expressions were tested through quantitative real‐time polymerase chain reaction using SYBR Green (04887352001, Roche), and the primers used are listed in Table 1. The results were normalized as ratio of target gene to GAPDH gene expression.

**Table 1:** Primers for Real‐Time PCR

Gene Name	Primer	Sequence
ANP‐Rat	Forward 5′‐3′	AAAGCAAACTGAGGGCTCTGCTCG
	Reverse 5′‐3′	TTCGGTACCGGAAGCTGTTGCA
β‐MHC‐Rat	Forward 5′‐3′	TCTGGACAGCTCCCCATTCT
	Reverse 5′‐3′	CAAGGCTAACCTGGAGAAGATG
GAPDH‐Rat	Forward 5′‐3′	GACATGCCGCCTGGAGAAAC
	Reverse 5′‐3′	AGCCCAGGATGCCCTTTAGT
ANP‐Mouse	Forward 5′‐3′	ACCTGCTAGACCACCTGGAG
	Reverse 5′‐3′	CCTTGGCTGTTATCTTCGGTACCGG
BNP‐Mouse	Forward 5′‐3′	GAGGTCACTCCTATCCTCTGG
	Reverse 5′‐3′	GCCATTTCCTCCGACTTTTCTC
β‐MHC‐Mouse	Forward 5′‐3′	CCGAGTCCCAGGTCAACAA
	Reverse 5′‐3′	CTTCACGGGCACCCTTGGA
Collagen Iα‐Mouse	Forward 5′‐3′	AGGCTTCAGTGGTTTGGATG
	Reverse 5′‐3′	CACCAACAGCACCATCGTTA
Collagen III‐Mouse	Forward 5′‐3′	CCCAACCCAGAGATCCCATT
	Reverse 5′‐3′	GAAGCACAGGAGCAGGTGTAGA
MMP2‐Mouse	Forward 5′‐3′	TTTGCTCGGGCCTTAAAAGTAT
	Reverse 5′‐3′	CCATCAAACGGGTATCCATCTC
GAPDH‐Mouse	Forward 5′‐3′	ACTCCACTCACGGCAAATTC
	Reverse 5′‐3′	TCTCCATGGTGGTGAAGACA

ANP indicates atrial natriuretic peptide; BNP, brain natriuretic peptide; MMP2, matrix metalloproteinase 2; β‐MHC, β‐myosin heavy chain; PCR, polymerase chain reaction.

### Western Blotting

Total proteins from mice ventricles and NRCMs were extracted in lysis buffer (720 μL radioimmunoprecipitation assay buffer, 20 μL phenylmethylsulfonyl fluoride, 100 μL Complete, 100 μL Phos‐stop, 50 μL NaF, 10 μL Na_3_VO_4_ in 1 mL lysis buffer) and the protein concentrations were assessed using the Pierce^®^ BCA Protein Assay Kit (23225; Thermo Fisher Scientific, Waltham, MA). Protein samples were separated by SDS‐PAGE (NP0301BOX; Invitrogen) and electrically transferred to polyvinylidene difluoride membranes (IPVH00010, Millipore Corporation). Membranes were blocked with 5% skimmed milk powder dissolved in Tris‐buffered saline with 0.1% Tween 20 about 60 minutes at room temperature, prior to overnight incubation with the indicated primary antibodies at 4°C. Then, the membranes were incubated with relevant secondary antibodies and visualized with a FluorChem E imager (Cell Biosciences, Santa Clara, CA). The specific protein expression levels were normalized as ratio of target protein to GAPDH transferred to the same polyvinylidene difluoride membrane.

### Statistical Analysis

Data were presented as the mean±SEM. A 2‐tailed Student *t* test was used to compare the difference between 2 groups, and differences among groups were assessed by 1‐way ANOVA followed by Bonferroni test (assuming equal variances) or Tamhane's T2 test (without the assumption of equal variances). Two‐factor ANOVA was performed to analyze differences by operation and genotype when we compared 4 groups. Statistical analysis was performed using SPSS software, version 16.0. *P*<0.05 was considered statistically significant.

## Results

### SNIP1 Expression Was Decreased in Hypertrophic Hearts and Cardiomyocytes

**Figure 1:**
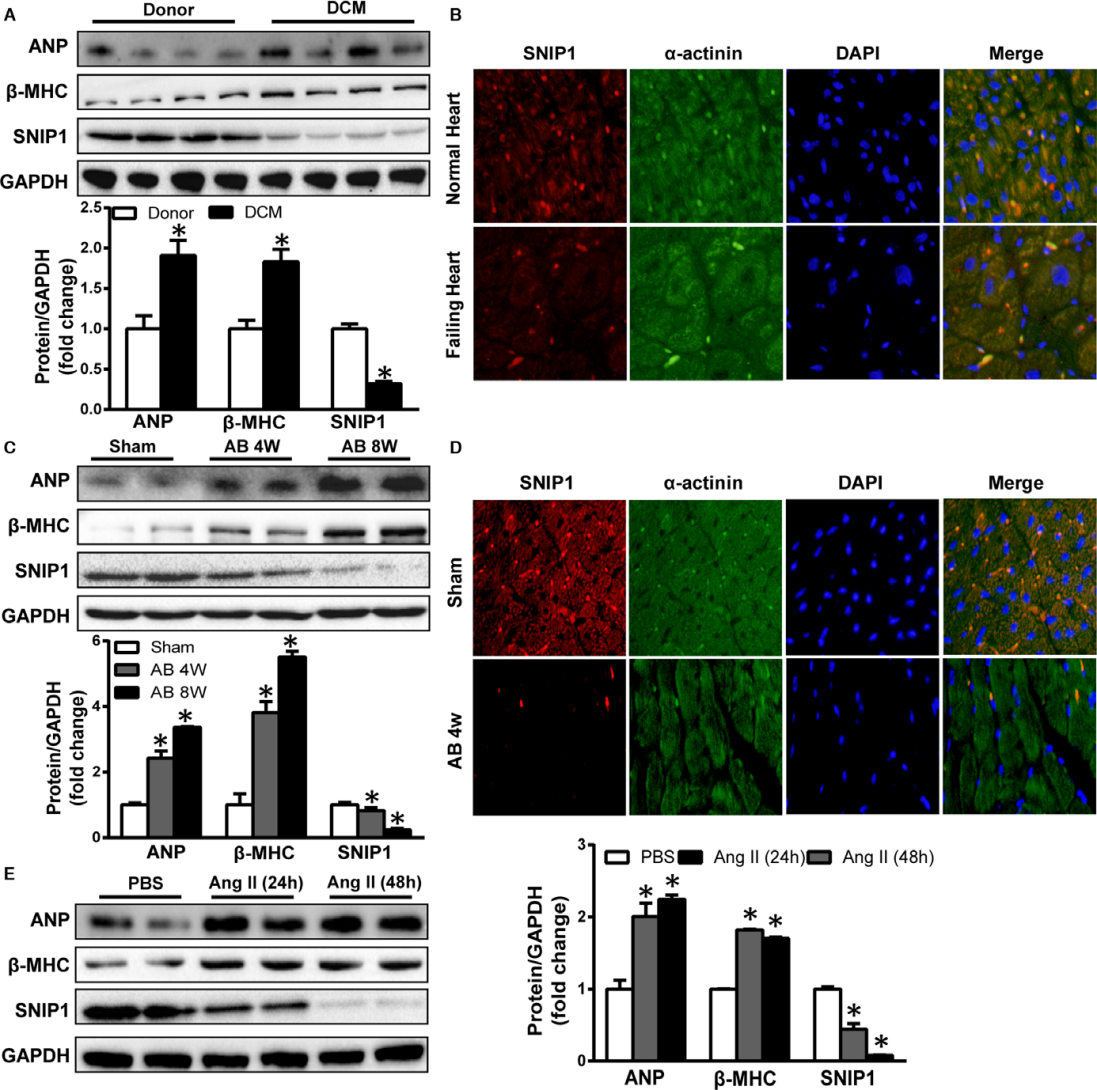
Smad nuclear interacting protein 1 (SNIP1) expression is downregulated in human dilated cardiomyopathy (DCM) hearts, hypertrophic murine hearts, and cardiomyocytes. A, Representative Western blot results and quantitative results of atrial natriuretic peptide (ANP), β‐myosin heavy chain (β‐MHC), and SNIP1 expression in both donor human hearts and DCM human hearts (n=6 samples per group). B, Double‐immunostaining of SNIP1 and α‐actininin in both donor human hearts and DCM human hearts (n=3 samples per group). DAPI, 4′,6‐diamidino‐2‐phenylindole. C, Representative Western blot results and quantitative results of ANP, β‐MHC, and SNIP1 expression 4 and 8 weeks after sham or aortic banding (AB) operation (n=4 mice per group). D, Double‐immunostaining of SNIP1 and α‐actininin 4 weeks after sham or AB operation (n=4 mice per group). E, Representative Western blot results and quantitative results of ANP, β‐MHC, and SNIP1 expression in neonatal rat cardiomyocytes treated with PBS or angiotensin II (Ang II, 100 nmol/L) for 24 and 48 hours (n=3 samples per group). Data are presented as the mean±SEM. *P<0.05 vs Donor, Sham, or PBS group.

To explore the potential involvement of SNIP1 in cardiac hypertrophy, we first assessed the SNIP1 expression in hypertrophic hearts and cardiomyocytes. As compared with normal hearts, the protein levels of 2 prominent hypertrophic markers, ANP and β‐MHC, were highly upregulated (Figure 1A), while expression of SNIP1 was significantly decreased in dilated cardiomyopathy hearts (Figure 1A and 1B). Consistently, cardiac SNIP1 expression was also dramatically reduced at 4 and 8 weeks after AB operation in mice (Figure 1C and 1D). According to the double‐immunostaining of SNIP1 and α‐actinin in the hearts (Figure 1B and 1D), cardiomyocytes were considered as the main cells expressing SNIP1. Interestingly, SNIP1 also expressed in cardiac fibroblasts isolated from wild‐type mice subjected to sham or AB operation, but without alteration (data not shown). Therefore, in an in vitro study, NRCMs were used and treated with Ang II, 100 nmol/L for 24 and 48 hours to induce hypertrophic responses, resulting in comparable trends of ANP, β‐MHC, and SNIP1 (Figure 1E). Together, these results indicated that the expression of SNIP1 was suppressed in cardiac hypertrophy.

### SNIP1 Elimination Exacerbated AB‐Induced Cardiac Hypertrophy and Fibrosis

**Figure 2:**
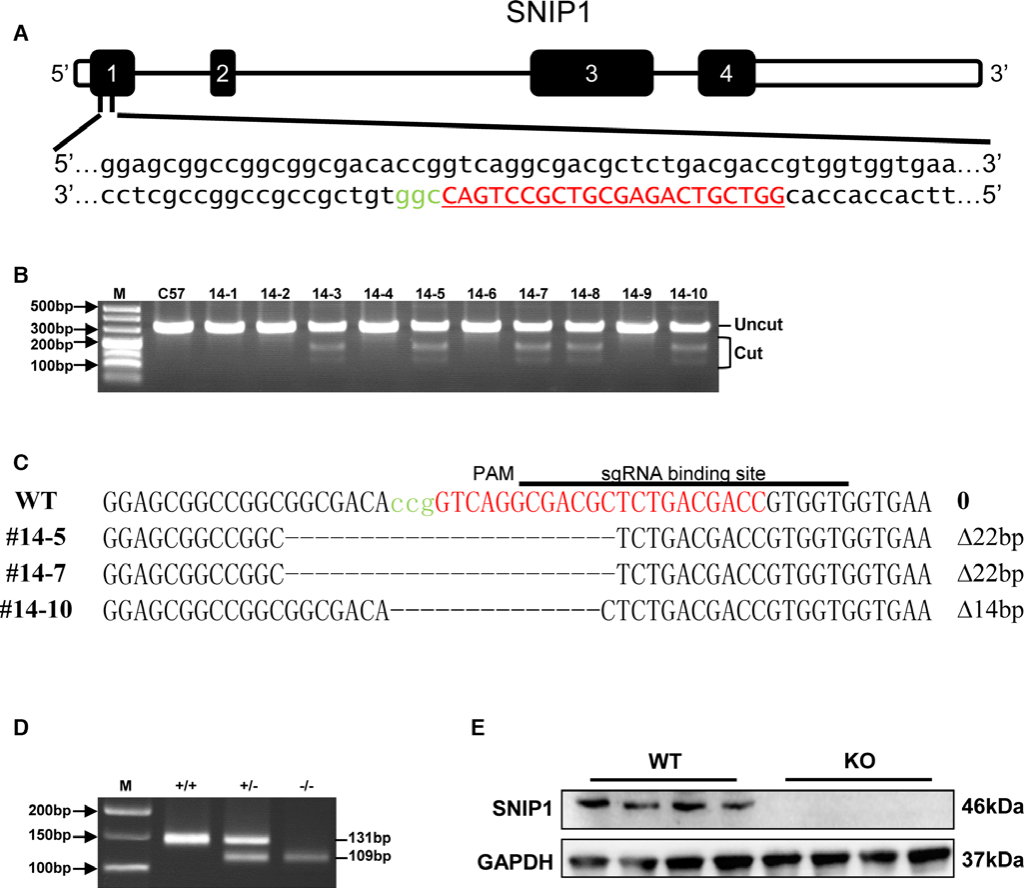
Schematic diagram of the construction of Smad nuclear interacting protein 1 (SNIP1) global knockout (SNIP1‐KO) mice using CRISPR‐Cas9 techniques. A, The single guiding RNA (sgRNA) chain was designed and constructed in exon 1. B, PCR results indicated that 5 of the 10 mice contained the cleavage products after microinjection. C, Mutant mice labeled #14‐5, #14‐7, and #14‐10 were sequenced. D, Heterozygous F1 offspring were interbred to establish the SNIP1‐KO mouse. E, Western blot analysis of SNIP1 expression in the heart in both wild type (WT) and KO mice (n=4 mice per group).

To tackle the question of whether SNIP1 is involved in AB‐induced cardiac hypertrophy, we generated global SNIP1 knockout mice using CRSIPR‐Cas9 techniques (Figure 2A through 2C), which were subjected to AB or sham operation. The excision of SNIP1 in SNIP1‐KO mice was confirmed by DNA and protein levels (Figure 2D and 2E). Of note, the SNIP1‐KO mice showed no apparent phenotypic character alteration in basal condition at 16 weeks of age.

**Figure 3:**
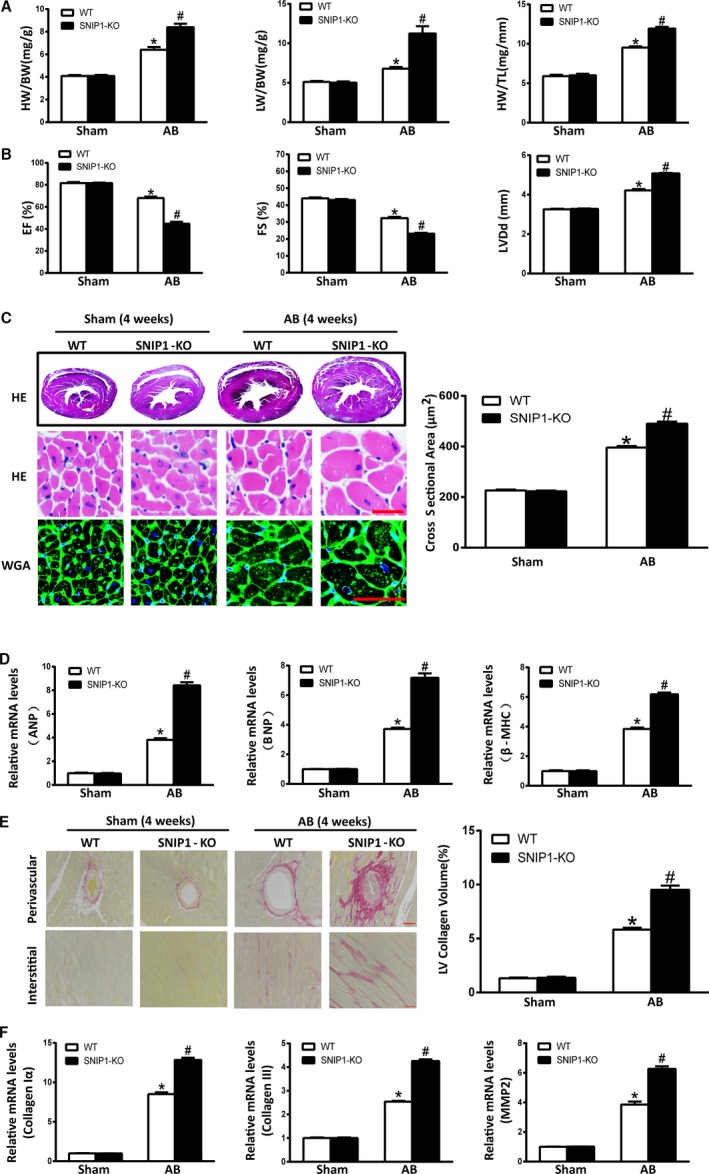
Smad nuclear interacting protein 1 (SNIP1) deficiency exacerbates aortic banding (AB)‐induced cardiac hypertrophy. A, Heart weight (HW)/body weight (BW), lung weight (LW)/BW, and HW/tibia length (TL) measured in wild‐type (WT) and SNIP1‐knockout (SNIP1‐KO) mice 4 weeks after sham or AB operation (Sham, n=10 mice per group; AB, n=12 mice per group). B, Results of ultrasound cardiogram test for WT and SNIP1‐KO mice at 4 weeks after sham or AB operation, including fraction shortening (FS), ejection fraction (EF), and left ventricular end‐diastolic diameter (LVDd) (Sham, n=5 mice per group; AB, n=7 mice per group). C, Left: Hematoxylin–eosin (HE) staining and wheat germ agglutinin (WGA) staining of WT and SNIP1‐KO mice hearts at 4 weeks after sham or AB operation (n=6 mice per group; scale bar, 50 μm). Right: Bar graph of calculated cross‐sectional area in the indicated groups (n>100 cells per group). D, The relative mRNA expression of atrial natriuretic peptide (ANP), brain natriuretic peptide (BNP), and β‐myosin heavy chain (β‐MHC) in WT and SNIP1‐KO mice at 4 weeks after sham or AB operation. E, Left: Picrosirius red staining of WT and SNIP1‐KO mice hearts at 4 weeks after sham or AB operation (n=6 mice per group; scale bar, 50 μm). Right: Bar graph of calculated left ventricular (LV) collagen volume in the indicated groups (n>25 fields per group). F, The relative mRNA expression of fibrotic markers collagen Iα, collagen III, and matrix metalloproteinase 2 (MMP2) in WT and SNIP1‐KO mice at 4 weeks after sham or AB operation. Data are presented as the mean±SEM. *P<0.05 vs WT/Sham group, ^#^ *P*<0.05 vs WT/AB group.

In the sham groups, the survival rates at 4 weeks were both 100% (n=10). After AB operation for 4 weeks, the survival rates reduced to 92.3% (n=12/13) in the wild‐type group, 80% (n=12/15) in the SNIP1‐KO group, and SNIP1‐KO mice showed more severe cardiac hypertrophy than wild‐type mice, as indicated by increased heart weight (HW)/body weight (BW), lung weight (LW)/BW, and HW/tibia length (TL) (Figure 3A and Table 2). Moreover, SNIP1 elimination promoted LV dilation and dysfunction as identified by results of ultrasound cardiogram test including FS, ejection fraction (EF), and LVDd values (Figure 3B and Table 2). Consistently, SNIP1 deficiency augmented pressure overload–induced cardiac hypertrophy as evidenced by HE and WGA staining (Figure 3C). Additionally, increased mRNA expression levels of cardiac hypertrophic markers ANP, brain natriuretic peptide, and β‐MHC mRNA expression were also determined in SNIP1‐KO mice after AB operation (Figure 3D). Subsequently, cardiac fibrosis was assessed. As expected, fibrosis in the interstitial and perivascular space was significantly increased in response to AB operation in SNIP1‐KO mice as compared with wild‐type mice (Figure 3E), as well as mRNA levels of fibrotic markers including collagen Iα, collagen III, and matrix metalloproteinase 2 (Figure 3F). Taken together, these data implied that SNIP1 deficiency accelerated cardiac hypertrophy and fibrosis under pressure overload.

**Table 2:** Parameters in WT and SNIP1‐KO Mice at 4 Weeks After Sham or AB Operation

Parameters	WT/Sham (n=10)	SNIP1‐KO/Sham (n=10)	WT/AB (n=12)	SNIP1‐KO/AB (n=12)	Two‐Factor ANOVA, *P* Value		
					Operation	Genotype	Interaction
BW, g	26.10±0.75	26.38±0.66	27.36±0.45	25.93±0.61	0.508	0.355	0.172
HW/BW, mg/g	4.09±0.09	4.09±0.10	6.41±0.23*	8.40±0.31*, †	<0.001‡	<0.001‡	<0.001‡
LW/BW, mg/g	5.11±0.12	5.02±0.16	6.79±0.21*	11.23±0.93*, †	<0.001‡	0.002‡	0.002‡
HW/TL, mg/mm	5.90±0.17	6.00±0.19	9.52±0.15*	11.93±0.22*, †	<0.001‡	<0.001‡	<0.001‡
HR, beats/min	541.40±17.45	558.20±21.58	533.86±5.14	528.71±6.64	0.156	0.647	0.392
IVSd, mm	0.64±0.02	0.68±0.00	0.75±0.01*	0.80±0.03*	<0.001‡	0.039‡	0.745
LVDd, mm	3.26±0.02	3.28±0.02	4.21±0.07*	5.07±0.03*, †	<0.001‡	<0.001‡	<0.001‡
LVPWd, mm	0.63±0.02	0.66±0.02	0.76±0.00*	0.76±0.00	<0.001‡	0.142	0.142
IVSs, mm	1.00±0.00	1.02±0.02	1.14±0.02*	1.19±0.03*	<0.001‡	0.226	0.654
LVDs, mm	1.82±0.02	1.86±0.02	2.86±0.06*	3.90±0.03*, †	<0.001‡	<0.001‡	<0.001‡
LVPWs, mm	1.02±0.02	1.02±0.02	1.19±0.01*	1.23±0.03*	<0.001‡	0.351	0.351
EF, %	81.60±0.93	81.60±0.40	68.00±1.35*	44.71±1.76*, †	<0.001‡	<0.001‡	<0.001‡
FS, %	44.00±0.55	43.00±0.63	32.29±0.75*	23.14±0.46*, †	<0.001‡	<0.001‡	<0.001‡

All values are presented as mean±SEM. AB indicates aortic banding; BW, body weight; EF, ejection fraction; FS, fractional shortening; HR, heart rate; HW, heart weight; IVSd, end‐diastolic interventricular septum thickness; IVSs, end‐systolic interventricular septum thickness; LVDd, left ventricular end‐diastolic diameter; LVDs, left ventricular end‐systolic diameter; LVPWd, end‐diastolic left ventricular posterior wall thickness; LVPWs, end‐systolic left ventricular posterior wall thickness; LW, lung weight; SNIP1‐KO, Smad nuclear interacting protein1‐knockout mice; TL, tibia length; WT, wild type.

*P<0.05 vs WT/sham group; ^†^ *P*<0.05 vs WT/AB group, using ANOVA;

^‡^ *P*<0.05, using Two‐Factor ANOVA.

### Cardiac SNIP1 Overexpression Protected Heart From Pressure Overload–Induced Cardiac Hypertrophy and Fibrosis

**Figure 4:**
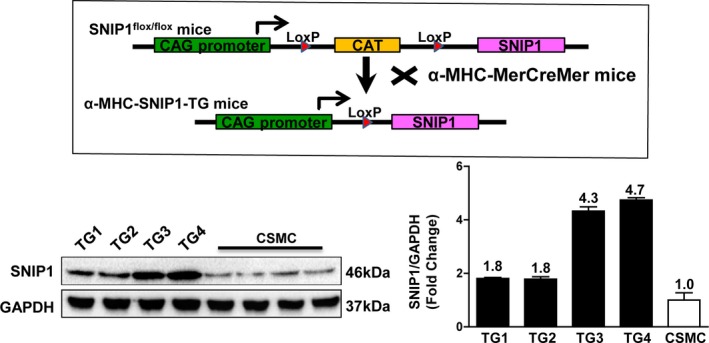
Schematic diagram of the construction of cardiac‐specific Smad nuclear interacting protein 1 (SNIP1) transgenic (TG) mice. Top: Schematic diagram of the construction of cardiac‐specific SNIP1‐TG mice. Bottom: Representative Western blot results and quantitative results of SNIP1 expression in the heart in the CAG‐CAT‐mSNIP1/α‐MHC‐MerCreMer mice without tamoxifen injection (CSMC) and SNIP1‐TG mice (n=4 mice per group).

To verify the role of SNIP1 in chronic pressure overload–induced cardiac hypertrophy and fibrosis, we created 4 lines of cardiac‐specific SNIP1 transgenic (TG) mice. Since protein expression of SNIP1 was about 4.7‐fold more in TG4 mice cardiac tissues than in CSMC mice (Figure 4), TG4 was chosen to perform our subsequent experiments. It is important to know that there were no functional or structural abnormalities in TG mice hearts as compared with nontransgenetic (CSMC) mice hearts under basal conditions at 16 weeks of age.

**Figure 5:**
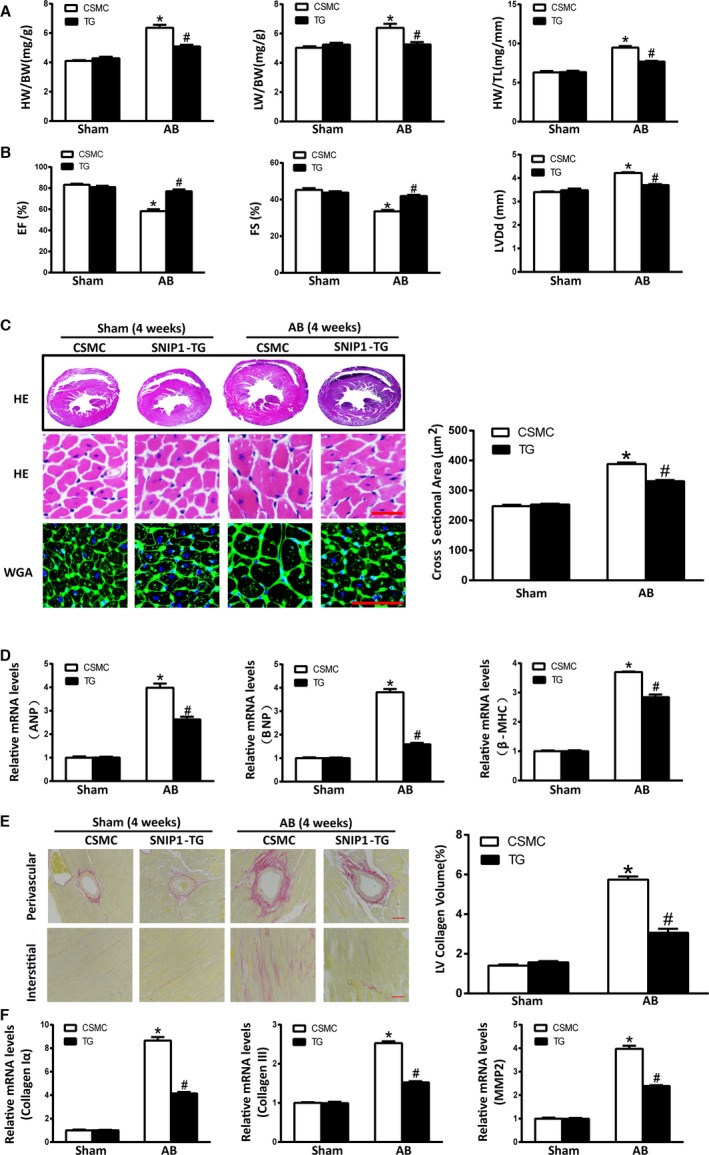
Cardiac‐specific overexpression of Smad nuclear interacting protein 1 (SNIP1) ameliorates pressure overload–induced cardiac hypertrophy and fibrosis. A, Heart weight (HW)/body weight (BW), lung weight (LW)/BW, and HW/tibia length (TL) measured in CAG‐CAT‐mSNIP1/α‐MHC‐MerCreMer mice without tamoxifen injection (CSMC) and SNIP1 transgenic (TG) mice at 4 weeks after sham or aortic banding (AB) operation (Sham, n=10 mice per group; AB, n=12 mice per group). B, Results of ultrasound cardiogram test for CSMC and SNIP1‐TG mice at 4 weeks after sham or AB operation, including fractional shortening (FS), ejection fraction (EF), and left ventricular end‐diastolic diameter (LVDd) (Sham, n=5 mice per group; AB, n=7 mice per group). C, Left: Hematoxylin–eosin (HE) staining and wheat germ agglutinin (WGA) staining of CSMC and SNIP1‐TG mice hearts at 4 weeks after sham or AB operation (n=6 mice per group; scale bar, 50 μm). Right: Bar graph of calculated cross‐sectional area in the indicated groups (n>100 cells per group). D, The relative mRNA expression of atrial natriuretic peptide (ANP), brain natriuretic peptide (BNP), and β‐myosin heavy chain (β‐MHC) in CSMC and SNIP1‐TG mice at 4 weeks after sham or AB operation. E, Left: Picrosirius red staining of CSMC and SNIP1‐TG mice hearts at 4 weeks after sham or AB operation (n=6 mice per group; scale bar, 50 μm). Right: Bar graph of calculated left ventricular (LV) collagen volume in the indicated groups (n>25 fields per group). F, The relative mRNA expression of fibrotic markers collagen Iα, collagen III, and matrix metalloproteinase 2 (MMP2) in CSMC and SNIP1‐TG mice at 4 weeks after sham or AB operation. Data are presented as the mean±SEM. *P<0.05 vs CSMC/Sham group, ^#^ *P*<0.05 vs CSMC/AB group.

The TG mice and their CSMC broods were subjected to AB or sham operation. In the sham groups, the survival rates at 4 weeks were both 100% (n=10). After AB operation for 4 weeks, the survival rates were 92.3% (n=12/13) in the CSMC group, and 100% (n=12) in the SNIP1‐TG group. Simultaneously, SNIP1‐TG mice mitigated cardiac hypertrophic phenotype as compared with CSMC mice after AB operation as proved by HW/BW, LW/BW, and HW/TL ratios (Figure 5A and Table 3), FS, ejection fraction and LVDd values (Figure 5B and Table 3), HE and WGA staining (Figure 5C), and mRNA levels of ANP, brain natriuretic peptide, and β‐MHC (Figure 5D). In addition, we examined the cardiac fibrosis in AB‐operated TG and CSMC mice. Our data of picrosirius red staining of cardiac paraffin sections and fibrotic markers mRNA expression (Figure 5E and 5F) revealed that SNIP1 overexpression could retard cardiac fibrosis induced by chronic pressure overload. In all, these evidences demonstrated that cardiac overexpressed SNIP1 attenuated AB‐driven cardiac hypertrophy.

**Table 3:** Parameters in CSMC and SNIP1‐TG Mice at 4 Weeks After Sham or AB Operation

Parameters	CSMC/Sham (n=10)	SNIP1‐TG/Sham (n=10)	CSMC/AB (n=12)	SNIP1‐TG/AB (n=12)	Two‐Factor ANOVA, *P* Value		
					Operation	Genotype	Interaction
BW, g	27.51±0.60	26.37±0.55	26.75±0.54	26.91±0.59	0.854	0.404	0.266
HW/BW, mg/g	4.10±0.06	4.29±0.10	6.37±0.20*	5.09±0.11*, †	<0.001‡	<0.001‡	<0.001‡
LW/BW, mg/g	5.03±0.10	5.25±0.13	6.39±0.28*	5.26±0.16†	0.001‡	0.021‡	0.001‡
HW/TL, mg/mm	6.30±0.16	6.33±0.18	9.47±0.19*	7.68±0.10*, †	<0.001‡	<0.001‡	<0.001‡
HR, beats/min	564.20±10.29	564.20±10.29	522.83±15.57	525.14±13.36*	0.007‡	0.932	0.932
IVSd, mm	0.66±0.02	0.68±0.00	0.78±0.02*	0.70±0.01†	<0.001‡	0.077	0.006‡
LVDd, mm	3.40±0.03	3.48±0.07	4.22±0.03*	3.70±0.04*, †	<0.001‡	<0.001‡	<0.001‡
LVPWd, mm	0.68±0.00	0.68±0.00	0.78±0.03*	0.71±0.02	0.001‡	0.087	0.087
IVSs, mm	1.00±0.00	1.02±0.02	1.18±0.03*	1.13±0.03*	<0.001‡	0.507	0.162
LVDs, mm	1.86±0.05	1.96±0.07	2.82±0.04*	2.14±0.05*, †	<0.001‡	<0.001‡	<0.001‡
LVPWs, mm	1.00±0.00	1.00±0.00	1.23±0.03*	1.10±0.03*, †	<0.001‡	0.008‡	0.008‡
EF, %	83.20±0.97	81.00±1.22	58.17±1.76*	77.00±1.76*, †	<0.001‡	<0.001‡	<0.001‡
FS, %	45.20±0.97	43.80±0.73	33.50±0.85*	41.86±0.67*, †	<0.001‡	<0.001‡	<0.001‡

All values are presented as mean±SEM. AB indicates aortic banding; BW, body weight; CSMC, CAG‐CAT‐mSNIP1/α‐MHC‐MerCreMer mice without tamoxifen injection; EF, ejection fraction; FS, fractional shortening; HR, heart rate; HW, heart weight; IVSd, end‐diastolic interventricular septum thickness; IVSs, end‐systolic interventricular septum thickness; LVDd, left ventricular end‐diastolic diameter; LVDs, left ventricular end‐systolic diameter; LVPWd, end‐diastolic left ventricular posterior wall thickness; LVPWs, end‐systolic left ventricular posterior wall thickness; LW, lung weight; SNIP1‐TG, Smad nuclear interacting protein1‐transgenic mice; TL, tibia length.

*P<0.05 vs CSMC/sham group; ^†^ *P*<0.05 vs CSMC/AB group, using ANOVA.

^‡^ *P*<0.05, using Two‐Factor ANOVA.

### SNIP1 Blocked Angiotensin II‐Induced Hypertrophic Response in Vitro

**Figure 6:**
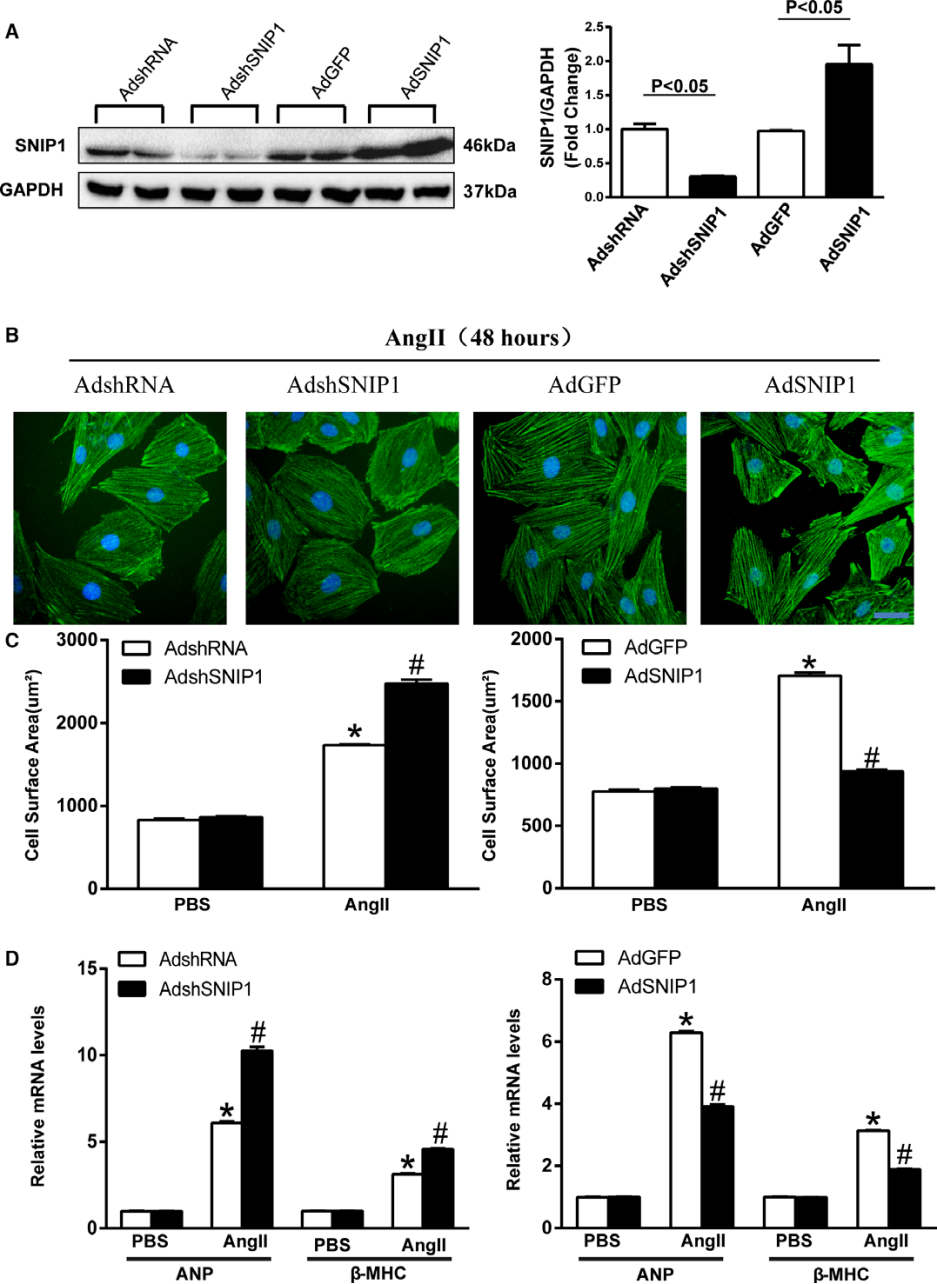
Smad nuclear interacting protein 1 (SNIP1) attenuates angiotensin II‐induced cardiomyocyte hypertrophy in vitro. A, Representative Western blot results and quantitative results of SNIP1 expression in neonatal rat cardiomyocytes (NRCMs) infected with AdshRNA, AdshSNIP1, AdGFP, and AdSNIP1. B, Representative images of NRCMs infected with AdshSNIP1 or AdSNIP1 (AdshRNA or AdGFP as control, respectively) in response to angiotensin II (Ang II, 100 nmol/L) for 48 hours (green, α‐actinin; blue, nuclei; scale bar, 20 μm). C, Bar graphs of calculated cell surface area in the indicated groups after 48 hours treatment with PBS or Ang II (n>50 cells per group). D, The relative mRNA expression of atrial natriuretic peptide (ANP) and β‐myosin heavy chain (β‐MHC) in the indicated groups after 48 hours treatment with PBS or Ang II. Data are presented as the mean±SEM. *P<0.05 vs AdshRNA/PBS group or AdGFP/PBS group, ^#^ *P*<0.05 vs AdshRNA/Ang II group or AdGFP/Ang II group.

To investigate the role of SNIP1 in cardiomyocyte hypertrophy in vitro, NRCMs were infected by either AdSNIP1 to overexpress SNIP1 or AdshSNIP1 to knockdown SNIP1 (Figure 6A). Then, NRCMs were stimulated with Ang II (100 nmol/L) for 48 hours, and subsequently subjected to immunostaining with α‐actinin antibody. Overexpression of SNIP1 suppressed Ang II‐driven enhancement of cell size, while knockdown of SNIP1 augmented the hypertrophic response to Ang II treatment (Figure 6B and 6C). In accordance with these findings, the increased expression of hypertrophic markers ANP and β‐MHC induced by Ang II stimulation was suppressed by SNIP1 overexpression and further elevated by SNIP1 attenuation (Figure 6D). Collectively, these data illustrated that SNIP1 inhibited cardiomyocyte hypertrophic effects induced by Ang II stimulation in vitro.

### SNIP1 Attenuated Cardiac Hypertrophy via Inhibition of NF‐κB Signaling Pathway

**Figure 7:**
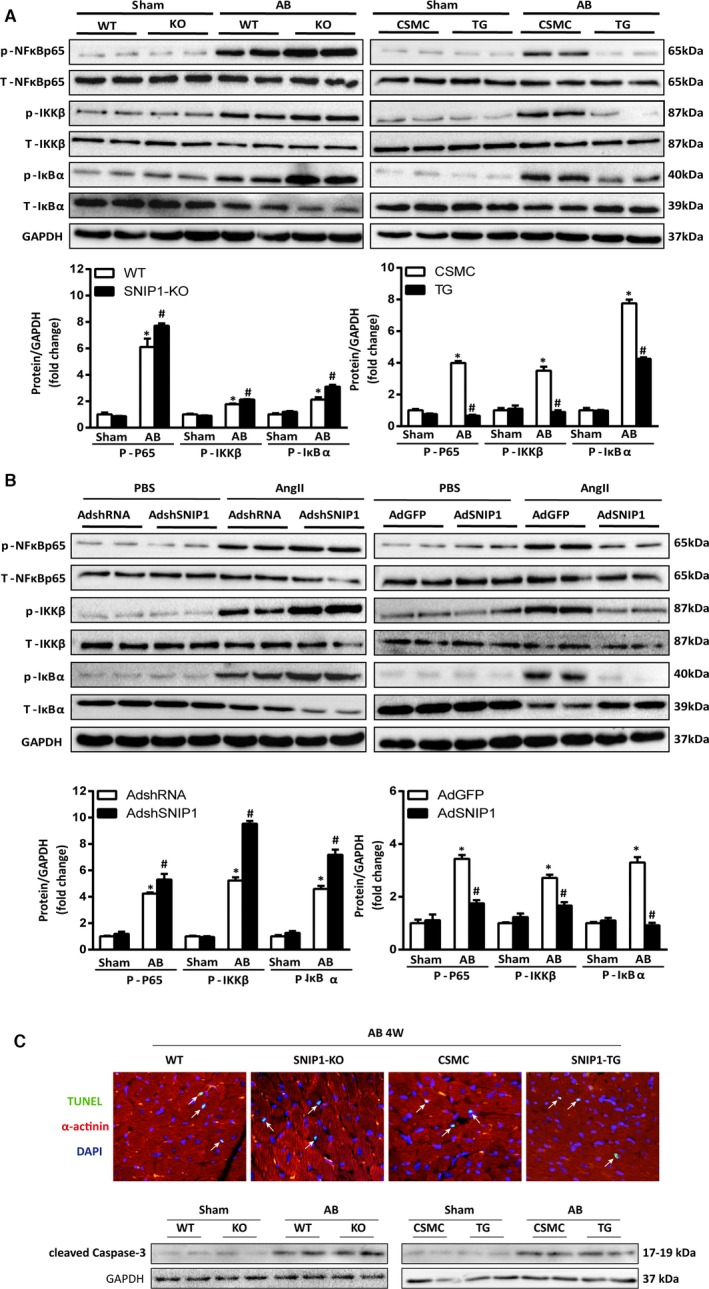
Smad nuclear interacting protein 1 (SNIP1) suppresses nuclear factor (NF)‐κB signaling in vivo and in vitro. A, Representative Western blot analysis and quantitative results of phosphorylated and total NF‐κB p65, inhibitor of κB kinase‐β (IKKβ), inhibitor of NF‐κB α (IκBα) expression in wild‐type (WT) and SNIP1‐knockout (SNIP1‐KO) mice or CAG‐CAT‐mSNIP1/α‐MHC‐MerCreMer mice without tamoxifen injection (CSMC) and SNIP1‐transgenic (SNIP1‐TG) mice 4 weeks after sham or aortic banding (AB) operation (n=6 mice per group). Data are presented as the mean±SEM. *P<0.05 vs WT/sham group or CSMC/sham group, ^#^ *P*<0.05 vs WT/AB group or CSMC/AB group. B, Representative Western blot analysis and quantitative results of phosphorylated and total NF‐κB p65, inhibitor of κB kinase‐β (IKKβ), inhibitor of NF‐κB α (IκBα) expression in AdshRNA, AdshSNIP1, AdGFP, AdSNIP1‐infected neonatal rat cardiomyocytes (NRCMs) treated with PBS or angiotensin II (Ang II, 100 nmol/L) for 48 hours. Data are presented as the mean±SEM. *P<0.05 vs AdshRNA/PBS group or AdGFP/PBS group, ^#^ *P*<0.05 vs AdshRNA/Ang II group or AdGFP/Ang II group. C, The TUNEL staining and representative Western blot analysis of cleaved Caspase‐3 in WT and SNIP1‐KO or CSMC and SNIP1‐TG mice 4 weeks after AB operation (n=4 mice per group). DAPI, 4′,6‐diamidino‐2‐phenylindole.

SNIP1 as a negative regulator of chronic pressure overload–induced cardiac hypertrophy prompted us to explore the mechanisms of SNIP1‐mediated antihypertrophic effects. In our present studies, the cardiac hypertrophy induced by AB operation was followed by the activation of NF‐κB signaling pathway (increased phosphorylation of NF‐κB p65, IKKβ, and IκBα), and this activation was significantly upregulated in the hearts of SNIP1‐KO mice and downregulated in TG mice hearts (Figure 7A). Consistent with the results in vivo, Ang II induced increase in phosphorylation of p65 (p‐NF‐κB p65), IKKβ (p‐IKKβ), and IκBα (p‐IκBα), as well as degradation of total IκBα (t‐IκBα) were further upregulated in AdshSNIP1‐infected NRCMs (Figure 7B). On the other hand, overexpression of SNIP1 in NRCMs by AdSNIP1 infection could inhibit Ang II‐induced activation of the NF‐κB p65 pathway (Figure 7B). As to apoptosis involved in cardiac remodeling, SNIP1 modification did not alter the pressure overload–induced apoptosis 4 weeks after AB operation (Figure 7C).

**Figure 8:**
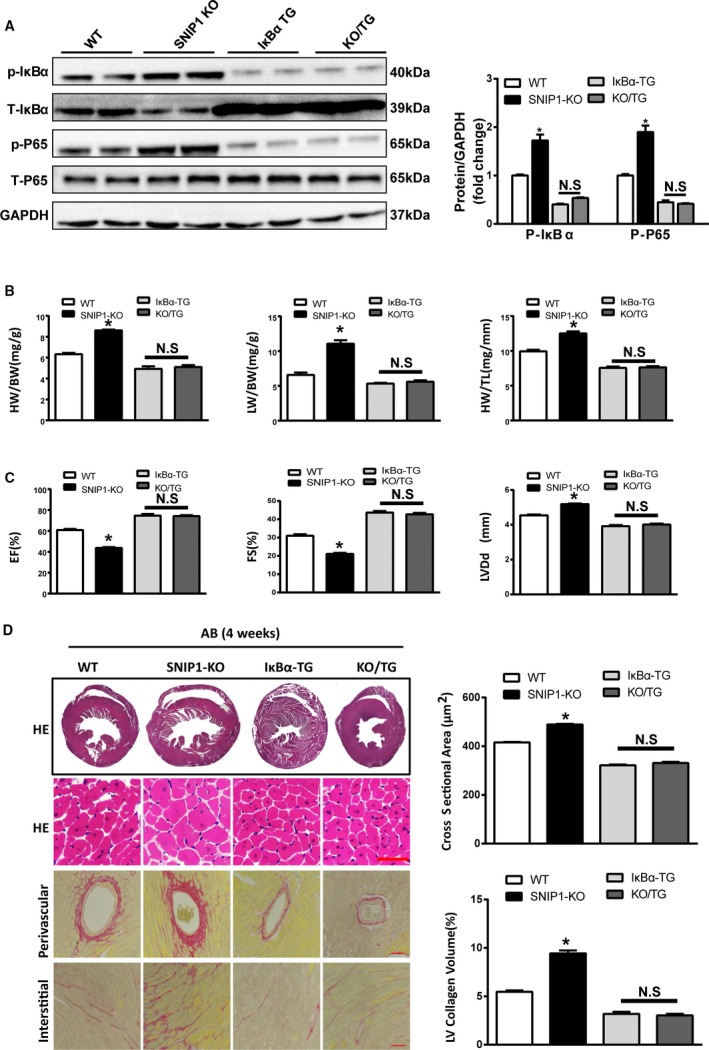
Overexpression of IκBα^S32A/S36A^ ameliorates the pernicious effects of Smad nuclear interacting protein 1 (SNIP1) deficiency on aortic banding (AB)‐induced cardiac hypertrophy. A, Representative Western blot analysis and quantitative results of phosphorylated and total NF‐κB p65, inhibitor of NF‐κB α (IκBα) expression in wild‐type (WT), SNIP1‐knockout (SNIP1‐KO), IκBα^S32A/S36A^ transgenic (IκBα‐TG), and SNIP1‐KO/IκBα‐TG (KO/TG) mice 4 weeks after AB operation (n=6 mice per group). B, Heart weight (HW)/body weight (BW), lung weight (LW)/BW, and HW/tibia length (TL) measured in the indicated mice 4 weeks after AB operation (n=10 mice per group). C, Results of ultrasound cardiogram test for the indicated mice at 4 weeks after AB operation, including fractional shortening (FS), ejection fraction (EF), and left ventricular end‐diastolic diameter (LVDd) (n=8 mice per group). D, Left: Hematoxylin–eosin (HE) staining and picrosirius red staining of the indicated mice hearts at 4 weeks after AB operation (n=5 mice per group; scale bar, 50 μm). Right: Bar graphs of calculated cross‐sectional area (n>100 cells per group) and left ventricular (LV) collagen volume (n>25 fields per group) in the indicated groups. Data are presented as the mean±SEM. *P<0.05 vs WT group; NS indicates no significant differences.

To further explore the relationship of the NF‐κB p65 pathway and SNIP1 in AB‐induced cardiac hypertrophy, we crossed the cardiomyocyte‐restricted overexpression of phosphorylation‐resistant IκBα (IκBα^S32A/S36A^) mice with SNIP1‐KO mice to generate IκBα (IκBα^S32A/S36A^)‐TG/SNIP1‐KO mice. Overexpression of IκBα (IκBα^S32A/S36A^) attenuated AB‐induced accumulation in phosphorylation of p65 (p‐NF‐κB p65), as well as the promoting effects of SNIP1‐KO on AB‐induced p‐NF‐κB p65 (Figure 8A). Moreover, our data showed that overexpression of IκBα^S32A/S36A^ obviously reversed the pro‐hypertrophic effects of SNIP1‐KO in AB‐induced cardiac hypertrophy, as evidenced by HW/BW, LW/BW, and HW/TL ratios (Figure 8B), FS, ejection fraction, and LVDd values (Figure 8C), and HE, WGA, and picrosirius red staining (Figure 8D). Therefore, these data suggested that the inhibition of NF‐κB signaling pathway might be a key factor of SINP1‐mediated protective effects on pressure overload–induced cardiac hypertrophy and fibrosis.

## Discussion

Our results suggest for the first time that SNIP1 plays a key role in mediating the development of pathological hypertrophy. SNIP1, as a new interactor of Smads, plays a critical role in cell proliferation, transformation of embryo fibroblasts, and immune regulation.^14^, ^15^ However, its role in pathological cardiac hypertrophy remains unclear. In the present study, we utilized gain‐of‐function and loss‐of‐function approaches to investigate the role of SNIP1 in the pathogenesis of cardiac hypertrophy. Our observations demonstrated that SNIP1 deficiency exacerbated pathological cardiac hypertrophy through activation of NF‐κB signaling in response to pressure overload, while cardiac‐specific overexpression of SNIP1 profoundly ameliorated pathological cardiac remodeling via inhibition of NF‐κB signaling. Moreover, cardiac‐specific overexpression of IκBα reversed the adverse effects of SNIP1 deficiency in AB‐induced pathological cardiac remodeling. Taken together, we proposed that SNIP1 exerted cardioprotective effects in pathological cardiac hypertrophy in part through NF‐κB signaling. Thus, SNIP1 upregulation may be a novel approach for the intervention and prevention of heart failure.

AB is recognized as the common and successful surgical model of chronic pressure overload–induced pathological cardiac hypertrophy,^11^, ^17^ and was used in the present studies. Here we report that SNIP1 was dramatically downregulated in human dilated cardiomyopathy hearts and AB‐induced murine hypertrophic hearts, as well as Ang II‐induced hypertrophic NRCMs. Importantly, a significant amelioration with improved cardiac function was observed in cardiac‐specific SNIP1 transgenic mice in response to chronic pressure overload. Accordingly, we assumed that SNIP1 was a protective factor in the progression of pathological cardiac remodeling. Hence, elucidation of the mechanisms underlying these characteristic phenomena related to SNIP1 will potentially further our understanding of cardiac hypertrophy and yield drugable targets for the intervention and prevention of cardiac hypertrophy.

SNIP1, first identified as a new interactor of Smads, could also interact with NF‐κB transcriptional factor p65/RelA.^16^ These transcriptional factors could also bind to the C/H1 domain of transcriptional coactivators CBP and p300.^13^ Therefore, SNIP1 could suppress the transcriptional activations of Smads and NF‐κB by blocking their interactions with CBP/p300.^13^, ^16^ Interestingly, SNIP1 not only attenuated phosphorylation and activation of NF‐κB p65, but also inhibited the activation of IKKβ and IκBα in the present study. Simultaneously, SNIP1 improved cardiac function and suppressed pathological cardiac hypertrophy and fibrosis. In addition, SNIP1‐KO/IκBα^S32A/S36A^‐TG mice rescued the deteriorative effects of SNIP1 knockout on AB‐induced cardiac remodeling and dysfunction. All these findings demonstrated that SNIP1, with the ability to regulate NF‐κB signaling, functioned as a protective and antihypertrophic factor, and was involved in the pathogenesis of cardiac remodeling.

It is well known that NF‐κB signaling is indispensable for the regulation of various cardiovascular diseases, especially for cardiac hypertrophy, fibrosis, and ventricular remodeling.^11^, ^12^, ^18^, ^19^, ^20^, ^21^, ^22^ Nevertheless, the role of NF‐κB signaling in the regulation of cardiac hypertrophy remains controversial. Some studies have revealed that NF‐κB activation is required for the progression of cardiac hypertrophy. For instance, IKK/NF‐κB activation in cardiomyocytes caused inflammatory cardiomyopathy and heart failure,^23^ while inhibition of NF‐κB signaling by a nondegradable IκBα decreased cardiac hypertrophy and dysfunction induced by Ang II stimuli and pressure overload.^11^, ^24^ Moreover, cardiac NF‐κB regulated angiogenesis and factors responsible for compensatory reaction to transverse aortic constriction–induced intracellular hypoxia, and cardiac‐specific deletion of p65 reduced the hypertrophic response after pressure overload stimulation.^19^ Consistently, in our present study, activation of NF‐κB signaling was observed in hypertrophic hearts, and SNIP1 overexpression suppressed the activation. More importantly, IκBα overexpression rescued the deteriorating effects of SNIP1 deficiency on AB‐induced cardiac hypertrophy. Therefore, the results as shown in this study hint heretofore new direct evidence that the inhibitory effects of SNIP1 on cardiac hypertrophy were partly owing to suppression of NF‐κB signaling. However, some other investigators have disputed the importance of NF‐κB in the pathogenesis of cardiac hypertrophy. Hikoso et al observed that IKKβ/NF‐κB signaling protected the cardiomyocytes against transverse aortic constriction–induced cardiac dilation and dysfunction through the attenuation of oxidative stress and c‐Jun N‐terminal kinase activation.^25^ Furthermore, NF‐κB inhibition by conditional deletion of the NF‐κB essential modulator/IKKγ augmented cardiac hypertrophy partly through modulation of oxidative stress.^26^ The main reason responsible for the conflicting results may be that NF‐κB has multiple divergent effects via various signaling pathways and finally either promote or suppress the pathogenic processes. Thus, further studies are needed to determine the therapeutic potential of SNIP1 for the treatment of pathological cardiac hypertrophy.

In order to rule out the side effects of SNIP1 on hypertrophic responses in other cell types except cardiomyocytes in the hearts, we used SNIP1‐shRNA and AdSNIP1 to interfere the expression of SNIP1 in cultured NRCMs. In accordance with the results in vivo, SNIP1 depletion in NRCMs aggravated Ang II‐mediated hypertrophic effect, while SNIP1 overexpression attenuated NRCMs hypertrophy in response to Ang II stimuli. Mechanically, Ang II‐induced NF‐κB activation increased in NRCMs infected by AdshSNIP1, whereas AdSNIP1 inhibited the NF‐κB activation in NRCMs. These findings further confirmed the protective effects of SNIP1 on cardiomyocytes against hypertrophy through inhibition of the NF‐κB signaling.

Cardiac hypertrophy is a complex and dynamic process, and involved in molecular diversity.^6^ Although NF‐κB signaling and SNIP1 have been confirmed to participate in various pathophysiological processes, our research cannot illuminate all the potential mechanisms that SNIP1 is involved in. Since single‐gene based either cell or animal research is likely to give very limited and usually biased understanding of cardiac hypertrophy, which is considered a multifactorial disease, there has been a lack of effort in decoding the detailed and full‐scale mechanisms of SNIP1 in cardiac hypertrophy. Thus, comprehensive investigations are needed to determine whether SNIP1 would be a reliable therapeutic target for cardiac hypertrophy and remodeling.

In conclusion, our findings provide the first direct evidence that SNIP1 is involved in mediating the adverse cardiac remodeling that occurs in response to hemodynamic overload and Ang II partly by regulation of NF‐κB signaling pathway. The beneficial effects of SNIP1 overexpression occurred in the improvement of cardiac structure and function. These findings suggest that targeting SNIP1 may offer novel hope in the prevention or intervention of cardiac remodeling in response to hypertrophic stimuli.

## Sources of Funding

This study was supported by grants from National Natural Science Foundation of China (No. 81470394, 81270256 to Ya‐wei Xu and No. 81270194 to Da‐chun Xu).

## Disclosures

None.
